# Current Professional Opinion

**Published:** 1906-05-26

**Authors:** 


### CURRENT PROFESSIONAL OPINION.

Attention has recently been attracted to the proceedings of two cults or creeds which, in spite of tolerably frequent censures by the juries of coroner's courts still continue to flourish in our midst. These cults are those of the Christian Scientist and of the Peculiar People. Since the conduct of individuals immediately concerned is still *sub judice*, comment upon these actual cases must be withheld, but it is permissible to note the wider bearings upon the public interest of the principle of conscientious objection in the matter of health. It will be quite apparent to anyone who reflects upon the subject that there is a very close analogy between the Christian Scientist and the conscientious objector to vaccination. In either case there is a manifest prejudice against what the majority of informed people consider to be the course dictated by wisdom, a prejudice so strong that those who hold it are quite content to endure for the sake of it the trouble of application for exemption, or the risk of public censure as the case may be. How far such a sacrifice for principle is admirable, or the reverse, is a question which from the point of view of philosophy leaves much opening for argument, although from the point of view of practice the answer seems plain enough. None the less, the law, by admitting the right of conscientious objection to vaccination, has opened a gate which can hardly be closed with any appearance of logic against a horde of other objections, provided only that they be backed by conscience.

The summer course of practical ophthalmology which was instituted at Oxford two years ago is to be repeated in July of the present year. The scheme has for its object the provision, within a limited space of time, of daily opportunities of gaining practical experience in the examination of the eye and in the recognition of ocular diseases. On the present occasion the course is to commence on Monday, July 9, and to terminate on Saturday, July 21. During the first week attention will be mainly given to the use of the ophthalmoscope and to the methods of detecting and estimating errors of refraction; and in the second week more specialised studies will be pursued. Clinical work and instruction will occupy some four hours of each day and will be conducted by the staff of the Oxford Eye Hospital. In addition, Professor Wm. Osler has promised to give a short series of clinical lectures at the Radcliffe Infirmary, and one of these is to be on the medical aspects of ophthalmology. The greater part of the routine instruction in ophthalmoscopic and refraction work will be undertaken by the University Reader in Ophthalmology, Mr. Robt. W. Doyne, and in the second part of the course a number of well-known ophthalmic surgeons will take part. Altogether the programme is a most complete and attractive one, and it will doubtless secure the repetition of former successes. The inclusive fee is five guineas and further particulars may be obtained from Mr. Robt. W. Doyne, 34 Weymouth Street, W.

The Medical Sickness and Accident Society, established in 1884, has made solid and gratifying progress. At the end of 1905 it had 2,429 members, an increase of ninety-three for the year, all of whom were male practitioners or licentiates in dental surgery practising in the United Kingdom. The funds increased by upwards of £9,000 during the year, making £196,284. The annual sickness premium income stands at £14,793, and the amount paid in sickness benefit was £11,132, being the largest sum yet disbursed in one year. Over £100,000 has been paid in sickness benefits during the last twenty-one years. It is very interesting to compare the results of the sickness fund with the experience of the Pension Fund for Nurses. The sickness fund now stands at £91,624, and shows a considerable gain during 1905. The results of the Pension Fund working are not nearly so favourable, owing to the proportionately larger number of policy-holders who receive benefit each year. This would point to the fact that the amount of sickness in the medical profession is not anything like so great as it is amongst nurses. It is fair to conclude that constant attendance on the sick leads to much more illness amongst the attendants than when such association is intermittent and irregular, as in the case of a visiting practitioner.

There has been a great deal of discussion lately in many parts of the world on anæsthesia by ethyl chloride. When it was introduced, a few years ago, it was stated to be a perfectly safe drug. The collection of some 30 or 40 deaths have somewhat alarmed administrators in this country. When these cases are analysed it is obvious in most of them why a fatality took place. Some were bad subjects for any sort of drug used as a narcotic, others were certainly due to faulty administration, apparatus, or experience. It is not sufficiently recognised that ethyl chloride narcosis requires not only a very careful choice of the patient, and care in administration, but also that, more than anything, is it necessary to have great experience to render it safe. The variable of its signs as to the measure of anæsthesia is well known, and in this consists the great difficulty in giving it. It is important to recognise that the patient becomes more deeply under its influence after the mask is removed; it is generally at this point that the fatal symptoms supervene, the surgeon being loth to begin when the patient does not seem to be quite under. When these points are carefully watched there appears to be more safety than with chloroform, and very little more danger than with gas. It is especially useful in out-patient hospital practice for operations lasting, say, five minutes, in which the effects of ether would not have passed off sufficiently soon for the patient to be able to go home. We advise general practitioners not to give an anæsthetic without another medical man being present, except, of couse, in the case of midwifery; but if a medical man in the country on his round comes across a case which he decides must have a short anæsthetic, he generally gives chloroform, then it would be better if he had experience of ethyl chloride to give it, as it is possible that he might have it with him, whereas he certainly would not have gas, portability not being one of its good points.

